# Molecular Signatures and Network Alterations Underlying GBM Progression and Recurrence

**DOI:** 10.3390/medicina62020336

**Published:** 2026-02-06

**Authors:** Andrea Pop Crisan, Cristina Ciocan, Radu Pirlog, Alexandru Necula, Darius Adin Al Hajjar, Lavinia-Lorena Pruteanu, Constantin-Ioan Busuioc, Deo Prakash Pandey, Aurel George Mohan, Cornelia Braicu, Ioana Berindan-Neagoe

**Affiliations:** 1 Department of Surgical Sciences, Faculty of Medicine and Pharmacy, University of Oradea, 410073 Oradea, Romania; 2 Department of Genomics, MEDFUTURE Institute for Biomedical Research, Iuliu Hațieganu University of Medicine and Pharmacy, 400347 Cluj-Napoca, Romania; 3 Département de Pathologie, Hôpitaux Universitaires Henri Mondor, AP-HP, 94010 Créteil, France; 4 INSERM U955, Université Paris Est Créteil, 94010 Créteil, France; 5 Clinic of Neurosurgery, Cluj County Emergency Clinical Hospital, 400347 Cluj-Napoca, Romania; 6 Faculty of Medicine, Iuliu Hațieganu University of Medicine and Pharmacy, 400347 Cluj-Napoca, Romania; 7 Department of Chemistry and Biology, Technical University of Cluj-Napoca, North University Center at Baia Mare, Faculty of Sciences, 430122 Baia Mare, Romania; 8 Department of Pathology, Saint Mary Clinical Hospital, 011172 Bucharest, Romania; busuioc.constantin@gmail.com; 9 Department of Pathology, Onco Team Diagnostic, 010719 Bucharest, Romania; 10 Department of Microbiology, Rikshospitalet, Oslo University Hospital, 0372 Oslo, Norway; deo.prakash@gmail.com; 11 CRESCO, Centre for Embryology and Healthy Development, University of Oslo, 0372 Oslo, Norway; 12 Doctoral School, Iuliu Hațieganu University of Medicine and Pharmacy, 400347 Cluj-Napoca, Romania; 13 Academy of Medical Sciences, 030171 Bucharest, Romania

**Keywords:** GBM, recurrent tumor, molecular pathways, gene networks, molecular signatures

## Abstract

*Background and Objectives*: Glioblastoma (GBM) is the most aggressive form of primary brain tumor, characterised by high recurrence rates and poor patient prognosis. This study aimed to identify gene-expression signatures and molecular networks associated with primary and recurrent GBM to better understand the biological mechanisms underlying tumor progression. *Materials and Methods*: Gene expression analysis of TCGA data was conducted to identify differentially expressed genes across tumor, recurrent, and normal brain tissues. Analysis of overlapping differentially expressed gene sets revealed both common and specific gene-expression profiles across the groups, highlighting genes potentially involved in GBM recurrence. Gene network and canonical pathway analyses were performed using Ingenuity Pathway Analysis (IPA) to identify key pathways and cellular functions altered in GBM. *Results*: Our data identified distinct molecular signatures in tumor, recurrent, and normal brain samples, highlighting dysregulated genes associated with cellular growth, proliferation, and movement. Transcriptomic stratification revealed progressive tumor- and recurrence-adapted states, with composite Tumor Scores (TS) and Recurrence Scores (RS) classifying samples into four classes: normal-like, proliferative, transitional, and recurrence-adapted tumor states. *Conclusions*: These findings provide insights into the signaling networks and biological mechanisms underlying GBM recurrence and may guide the identification of potential therapeutic targets to improve the management of this malignancy.

## 1. Introduction

GBM (GBM) is an incurable malignancy, accounting for approximately 15% of all intracranial neoplasms and nearly half of malignant gliomas [1,2,3]. Despite maximal surgical resection followed by concurrent radiotherapy and temozolomide chemotherapy, the median survival of GBM patients remains around 12 to 15 months, with fewer than 5% of patients surviving beyond five years, a fact unchanged for two decades [2,4]. Moreover, tumor recurrence is almost inevitable, often occurring within months of initial therapy, and recurrent GBM is typically more invasive and resistant to conventional treatments [2,5,6].

In recent years, high-throughput molecular profiling technologies, such as microarray-based gene expression analysis, next-generation sequencing, single-cell RNA sequencing, and proteomics, have revolutionized clinical research. Transcriptomic profiling has enabled researchers to classify GBM into distinct molecular subtypes (proneural, classical, and mesenchymal), each associated with unique signalling pathways, therapeutic responses, and prognostic outcomes [7]. These studies have identified key driver genes and dysregulated pathways involved in tumorigenesis, including alterations in EGFR, PTEN, TP53, and IDH1/2, as well as activation of signalling cascades such as PI3K/AKT/mTOR, MAPK, and NF-κB [5,8,9,10].

Gene expression profiling is a critical tool for elucidating the altered molecular mechanisms that differentiate primary from recurrent GBM [11]. Transcriptomic studies have revealed that recurrent GBMs often acquire novel mutations and gene-expression changes that promote enhanced cell migration, invasion, angiogenesis, and immune evasion [10,12,13]. Notably, recurrent tumors frequently overexpress genes involved in extracellular matrix remodelling, such as MMP2, MMP9, and integrins, as well as inflammatory mediators like IL-6 and STAT3, which support tumor progression and a pro-tumor microenvironment [5,14]. Stem cell-associated markers, including SOX2 and CD44, are also upregulated, reflecting an enrichment of tumor-initiating cells that drive therapy resistance [15]. In addition, recurrent GBMs exhibit elevated expression of drug-resistance-related genes that contribute to resistance to alkylating agents, chemotherapeutics, and apoptosis-inducing treatments [16]. Collectively, these molecular adaptations illustrate the plasticity of GBM cells under therapeutic pressure and underscore the challenges in achieving durable responses [5,10,12].

**Figure 1:**
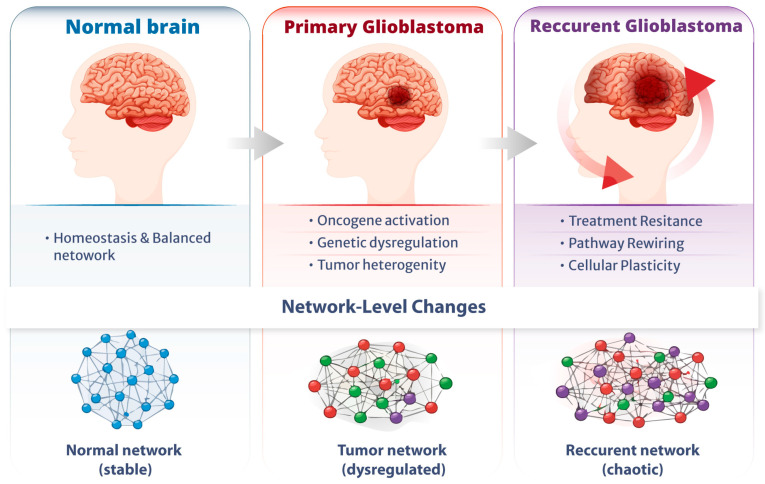
Network-level transcriptomic evolution in GBM. Schematic overview of GBC progression from normal brain to primary and recurrent disease. Normal tissue exhibits a stable, homeostatic gene regulatory network. Primary GBM is characterized by oncogenic activation, transcriptional dysregulation, and tumor heterogeneity, which together result in a restructured tumor network. Recurrent GBM exhibits therapy-driven network rewiring, cellular plasticity, and treatment resistance, resulting in a highly complex and unstable regulatory state.

Despite significant progress in molecular characterization of GBM, the biological foundations of recurrence mechanisms remain incompletely understood. Profiling gene expression and molecular networks in primary and recurrent GBM can reveal critical pathways underlying tumor adaptation and therapeutic failure. This study aims to identify and compare gene-expression signatures and signalling networks between primary and recurrent GBM tissues. Using Ingenuity Pathway Analysis (IPA), we systematically explored disease-related pathways, molecular and cellular functions, and gene networks. Furthermore, survival data from UALCAN datasets were integrated to assess the prognostic relevance of the identified genes. By combining gene expression profiling with pathway analysis, our work provides a comprehensive understanding of the molecular mechanisms underlying GBM progression and recurrence, emphasizing network transcriptomic evolution as shown in Figure 1.

## 2. Materials and Methods

**TCGA Data Collection.** One hundred fifty-four GBM samples, along with five matched normal tissue samples and thirteen recurrent tumor tissues, were obtained from the Cancer Genome Atlas (TCGA) database (http://firebrowse.org/, accessed on 20 November 2024). Table 1 provides detailed clinical information for the patients included in this study.

Gene Spring data analysis of the TCGA dataset was conducted to provide a baseline reference for non-neoplastic transcriptional programs and to highlight tumor-associated changes in gene expression relative to normal brain. To address potential batch effects in the TCGA data, samples were processed and normalized using standard GeneSpring (Agilent Technologies, V15.5) workflows, including background correction and quantile normalization. These procedures help ensure comparability across samples and minimize technical variability. Differential expression analysis was conducted using a fold-change (FC) threshold of ≥1.5 using the Benjamini–Hochberg correction. Only genes meeting both the FC and adjusted *p*-value criteria were considered significantly differentially expressed.

**Gene network analysis in GBM.** Gene network analysis was conducted using Ingenuity Pathway Analysis (IPA) software (Ingenuity Systems, Redwood City, CA, USA). Altered genes identified in GBM were imported into Ingenuity Pathway Analysis (IPA, QIAGEN, Hilden, Germany) to generate connectivity-based interaction networks using standard IPA settings. Networks were constructed based on known molecular interactions curated in the IPA knowledge base, with network scores calculated using a right-tailed Fisher’s exact test to assess the probability of the observed gene overlap occurring by chance. Each network score reflects the degree of enrichment of the input gene set within the IPA database. Canonical pathway analysis was performed to identify significantly enriched pathways, with significance determined using a −log10(*p*-value) threshold derived from Fisher’s exact test. Pathways with adjusted *p*-values meeting standard IPA significance criteria were considered relevant to the dataset.

Additional analyses were conducted to assess overlaps among GBM-associated gene sets related to epithelial–mesenchymal transition (EMT) and drug resistance using the Venn Diagram online tool. Genes common to these overlapping sets were subsequently used to explore functional connectivity and interaction patterns within the identified networks.

Protein–protein interaction (PPI) networks were generated using the STRING database (v12.0, accessed 12 November 2025) [17]. Default STRING settings were applied, including a medium confidence interaction threshold (combined score ≥ 0.4) and integration of evidence from experimental data, curated databases, co-expression, text mining, and predicted interactions.

**Gene expression-based scoring and tumor classification using tumor score (TS) and recurrence score (RS).** Normalized gene expression data from normal brain, primary glioblastoma, and recurrent glioblastoma samples were analysed. Lowly expressed and non-informative genes were filtered out before analysis. Differential expression analysis was performed to identify tumor-associated genes (tumor vs. normal) and recurrence-associated genes (recurrent vs. primary tumors). These gene sets were used to construct a TS and an RS, respectively. For each sample, gene expression values were standardized, and composite scores were calculated as the mean expression of upregulated genes minus the mean expression of downregulated genes. Samples were initially classified as normal-like (Class 1) or tumor-like (Class 2) using the TS.

RS subsequently stratified tumor samples into primary tumor—proliferative core (Class 2), primary tumor—transitional (Class 3), or recurrence-adapted aggressive tumors (Class 4), reflecting increasing transcriptional activity associated with recurrence. TS and RS were constructed from differential expression signatures. For each contrast (tumor vs. normal; recurrent vs. primary), genes significantly upregulated and downregulated (FDR < 0.05) were selected, and expression values were z-score standardized. For each sample, scores were calculated as the mean z-score of upregulated signature genes minus the mean z-score of downregulated signature genes. All statistical analyses were performed using R, with significance defined as *p* < 0.05. We combined multiple up- and down-regulated genes using a composite score to provide a robust transcriptional program and reduce the impact of noise from individual genes, particularly in heterogeneous tumors such as glioblastoma. Limitations of this study include the small number of recurrent samples. This can affect the solidity of the recurrence-associated signature, and the classification has not yet been validated in an independent cohort. External validation in larger datasets will be needed to confirm the extent to which our study findings can be extrapolated to a larger population.

## 3. Results

**Figure 2:**
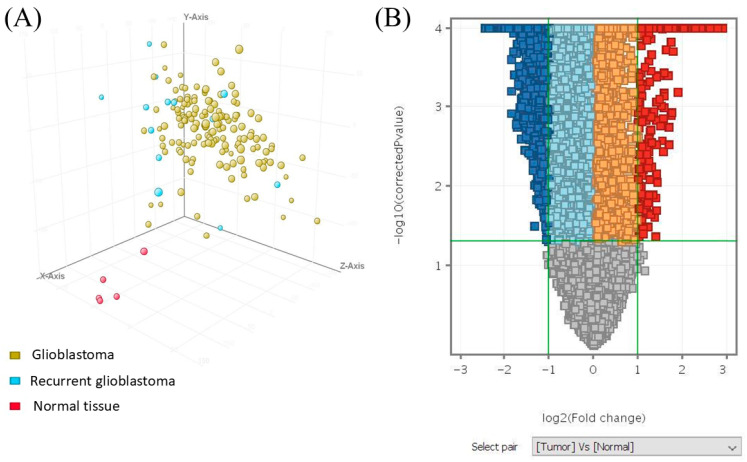
**Global gene expression differences between GBM and normal brain tissue.** (**A**) Principal component analysis (PCA) showing separation of GBM and normal brain samples based on global transcriptomic profiles. (**B**) Volcano plot illustrating differential gene expression between GBM and normal brain tissue. Red and blue denote significantly upregulated and downregulated genes, respectively, while grey indicates non-significant genes.

**Global transcriptomic differences between GBM and normal brain tissue.** Unsupervised principal component analysis revealed clear segregation between GBM, and normal brain samples based on global gene expression profiles, indicating distinct transcriptomic states associated with malignant transformation (Figure 2A). The first three principal components accounted for 12.95%, 8.13%, and 7.47% of the total variance, respectively, and GBM samples formed a distinct cluster from normal brain tissue.

Volcano plot analysis identified many significantly upregulated and downregulated genes meeting both fold-change and statistical significance thresholds, highlighting widespread alterations in gene expression accompanying GBM development (Figure 2B).

**Figure 3:**
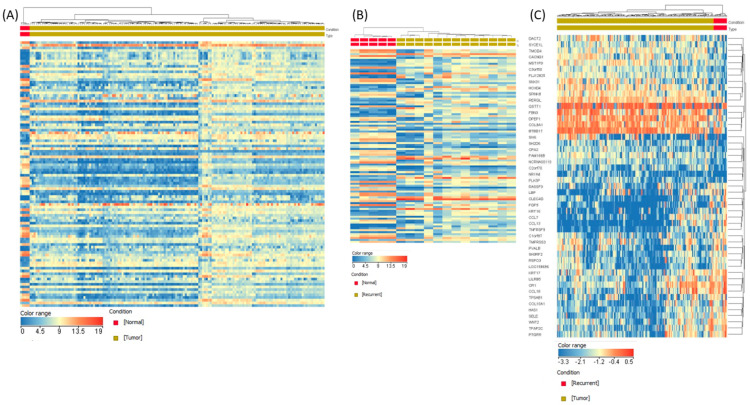
Heatmap of gene expression data in GBM. (**A**) Primary tumor versus normal brain tissue, (**B**) Recurrent tumor versus normal brain tissue, (**C**) Recurrent tumor versus primary tumor. Red or blue colors indicate differentially up- or downregulated genes, respectively.

**Gene expression alterations in GBM**. When comparing gene expression in tumour samples to peritumoral normal tissue, the analysis revealed a high number of differentially expressed genes (3051 downregulated and 2906 upregulated, Table S1). In the case of recurrent cases versus normal tissue, 4644 genes had an altered expression level (2388 downregulated and 2256 overexpressed, Table S2), in the case of recurrent tumor versus primary tumor, 607 genes had an altered expression level (99 downregulated and 508 overexpressed, Table S3). The heatmap graphical representation of the analyzed groups is presented in Figure 3.

**Figure 4:**
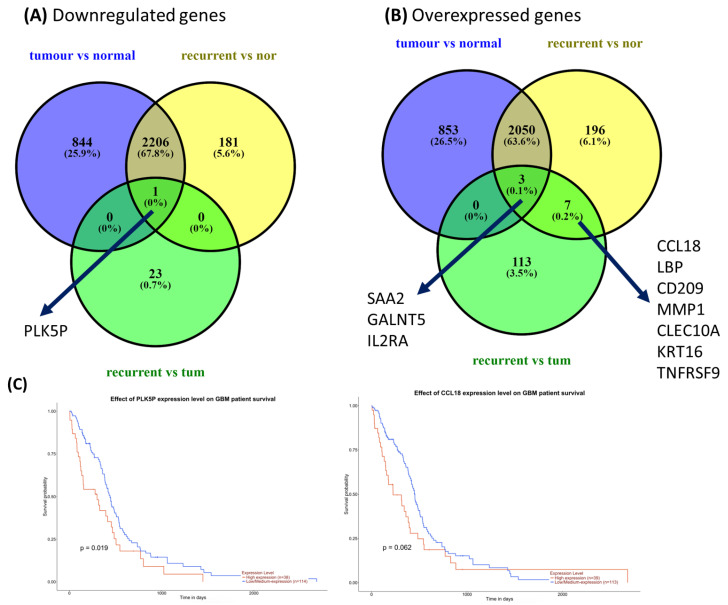
Shared and distinct gene expression signatures in GBM. Venn diagrams depicting common and group-specific (**A**) downregulated genes and (**B**) upregulated genes across GBM groups. (**C**) Overall survival analysis based on gene expression levels using UALCAN datasets.

**Shared and distinct gene expression signatures associated with GBM progression and survival**. Comparative analysis across GBM groups identified both shared and group-specific gene expression signatures, reflecting conserved and context-dependent transcriptional programs during disease progression (Figure 4). Venn diagram analysis revealed both overlapping and unique sets of downregulated and upregulated genes between primary and recurrent tumors (Figure 4A,B). Importantly, survival analysis of the UALCAN dataset showed that expression levels of selected genes were significantly associated with overall survival, linking these transcriptional alterations to clinical outcomes and supporting their biological and prognostic relevance (Figure 4C).

**Gene network and pathway alterations associated with GBM recurrence.** To gain mechanistic insight into the transcriptional differences observed between GBM subgroups, gene network and pathway analyses were performed using IPA, for the top 25 overexpressed and the top 25 downregulated genes. Integration of differential expression patterns with biological interpretation enabled classification of samples into four transcriptional states ranging from normal-like to recurrence-adapted aggressive tumors, each characterized by distinct marker gene signatures reflecting neuronal identity, proliferative activity, lineage transition, and immune–extracellular matrix remodeling (Table 2).

Disease and function annotation demonstrated that comparisons between tumor and normal tissues, as well as recurrent tumor and normal tissues, were dominated by cancer-related processes, organismal injury, endocrine and neurological disorders, and pathways linked to cellular growth, proliferation, and development (Table 2). In contrast, the recurrent versus primary tumor comparison highlighted a more focused set of alterations, with enrichment for inflammatory response, cellular movement, and cell–cell signalling, suggesting adaptive transcriptional programs associated with tumor relapse.

Network-level analysis further underscored these differences, identifying high-scoring interaction networks related to DNA replication and repair, developmental programs, immune regulation, and metabolic processes across the analyzed groups (Table 3). Notably, recurrent tumors showed prominent enrichment of immune-mediated and extracellular matrix-associated networks, consistent with a shift toward a more aggressive and adaptive phenotype.

**Figure 5:**
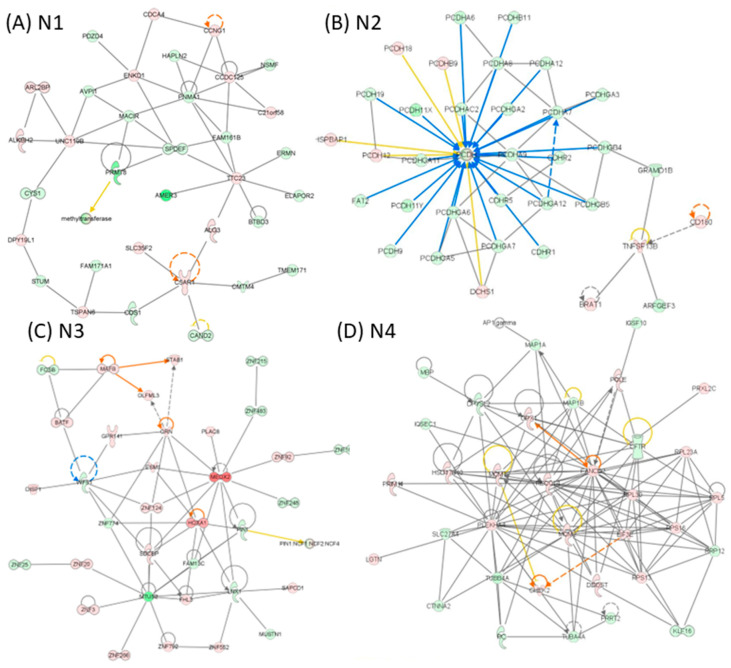
IPA network analysis of altered gene expression patterns in GBM. (**A**) N1, (**B**) N2, (**C**) N3, and (**D**) N4 networks for the altered gene expression list for the group tumour versus normal; red indicates upregulated genes, while green denotes downregulated genes. Orange lines represent predicted activation, and blue lines represent predicted inhibition. Genes that lack a clear directional change prediction are shown in white. The lines connecting genes illustrate various relationships, as explained in the legend.

**Figure 6:**
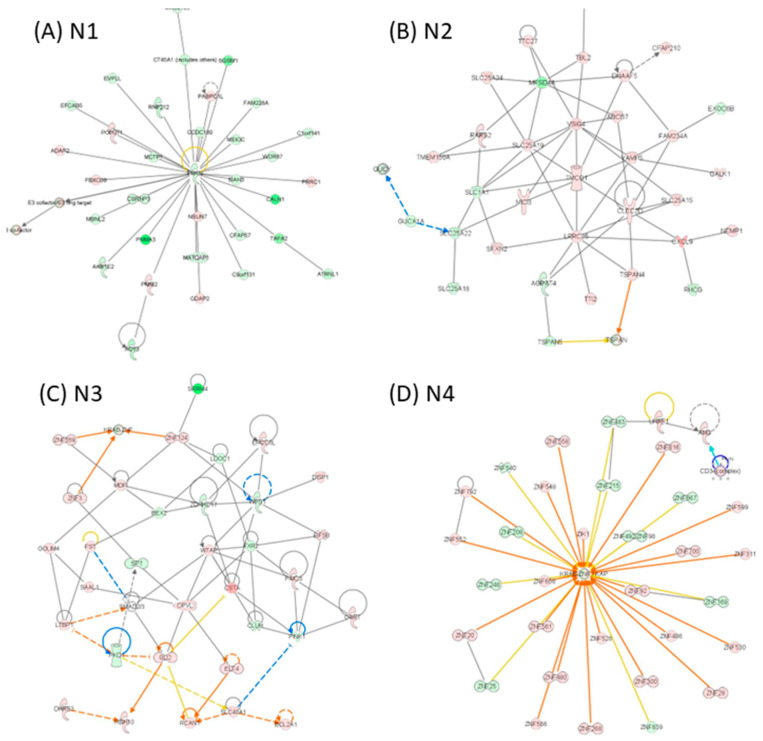
IPA network analysis of altered gene expression patterns in group recurrent GBM versus normal. Network (**A**) N1, (**B**) N2, (**C**) N3, and (**D**) N4 for the altered gene expression list for the group recurrent versus normal; red indicates upregulated genes, while green denotes downregulated genes. Orange lines represent predicted activation, and blue lines represent predicted inhibition. Genes that lack a clear directional change prediction are shown in white. The lines connecting genes illustrate various relationships, as explained in the legend.

**Figure 7:**
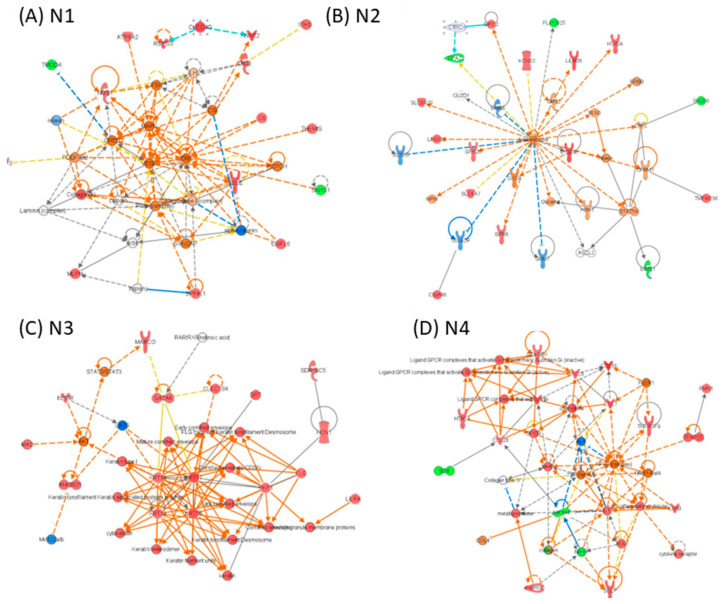
IPA network analysis of altered gene expression patterns in recurrent versus non-recurrent GBM. (**A**) N1, (**B**) N2, (**C**) N3, and (**D**) N4 network for the altered gene expression list for the group recurrent versus tumour; red indicates upregulated genes, while green denotes downregulated genes. Orange lines represent predicted activation, and blue lines represent predicted inhibition. Genes that lack a clear directional change prediction are shown in white. The lines connecting genes illustrate various relationships, as explained in the legend.

**Network-level reprogramming distinguishes primary and recurrent GBM.** IPA network reconstruction revealed distinct and context-specific gene interaction networks across GBM disease states (Figure 5, Figure 6 and Figure 7). In tumor versus normal tissue, the top-ranked networks were dominated by pathways related to DNA replication and repair, developmental programs, gene expression control, and cancer-associated signalling, reflecting widespread oncogenic reprogramming in primary tumors (Figure 5). In recurrent tumors compared with normal tissue, network architecture shifted toward pathways associated with immune response, cellular maintenance, metabolism, and stress adaptation, consistent with transcriptional remodeling accompanying tumor recurrence (Figure 6). Direct comparison of recurrent versus primary tumors uncovered a more focused set of networks enriched for inflammatory signalling, immune cell trafficking, cellular movement, and extracellular matrix interactions, highlighting adaptive programs linked to tumor progression and therapeutic resistance (Figure 7). Together, these network-level analyses illustrate progressive transcriptional rewiring during GBM evolution from primary disease to recurrence, reflecting adaptive responses to therapeutic pressure.

**Figure 8:**
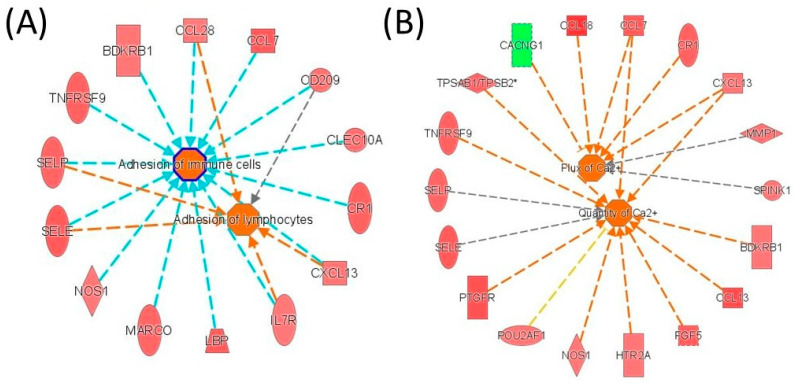
IPA network analysis of altered gene expression in recurrent versus non-recurrent GBM. IPA-derived networks illustrating (**A**) cell–cell adhesion and signalling and (**B**) calcium ion flux pathways in recurrent versus non-recurrent tumors. Red and green indicate upregulated and downregulated genes or regulatory elements, respectively.

**Increased cell–cell communication and calcium signalling characterize recurrent GBM.** IPA network analysis comparing recurrent and non-recurrent GBM revealed coordinated activation of pathways involved in cell-to-cell communication and intracellular signalling (Figure 8). Recurrent tumors exhibited increased activation of networks associated with cellular adhesion and tumor–microenvironment interactions, suggesting enhanced intercellular communication that may contribute to aggressive behavior and therapeutic resistance (Figure 8A). In parallel, activation of calcium flux signalling networks was observed, indicating dysregulation of calcium homeostasis as a potential driver of tumor cell proliferation, survival, and adaptive stress responses in recurrent disease (Figure 8B).

**Figure 9:**
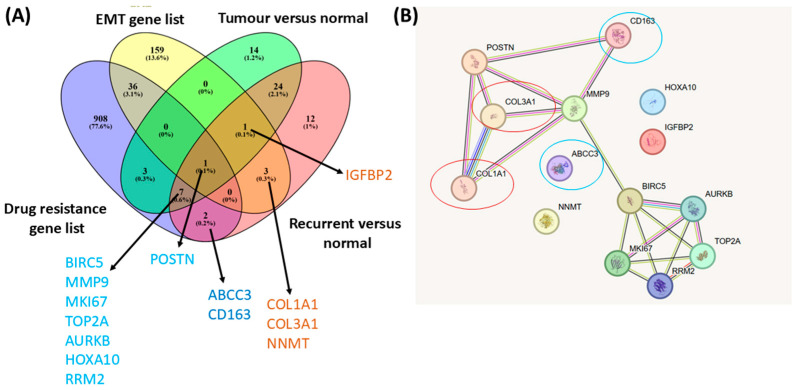
Overlap between GBM gene expression signatures, epithelial–mesenchymal transition (EMT), and drug resistance. (**A**) Venn diagram illustrating shared and specific gene expression signatures between GBM gene sets (tumor versus normal and recurrent versus normal) and curated EMT and drug resistance gene lists. (**B**) STRING-based protein–protein interaction network of overlapping genes identified specifically in the recurrent versus normal comparison. Genes associated with drug resistance are highlighted in blue, while EMT-related genes are highlighted in red.

**Convergence of EMT and drug resistance gene networks in recurrent GBM.** Comparative analysis of GBM gene expression signatures with curated epithelial–mesenchymal transition (EMT) and drug resistance gene sets revealed both shared and recurrence-specific molecular programs (Figure 9). Venn diagram analysis identified genes uniquely associated with recurrent GBM, including *COL1A1*, *COL3A1*, and *NNMT*, which are linked to drug resistance, and *IGFBP2*, associated with EMT, while *POSTN* emerged as a shared mediator connecting both processes (Figure 9A). Network-based interaction analysis further demonstrated extensive connectivity between EMT- and drug resistance-related genes, highlighting coordinated activation and potential crosstalk between these pathways in recurrent tumors (Figure 9B). Together, these findings suggest that EMT and drug resistance programs are functionally intertwined and may cooperatively promote GBM progression and recurrence.

**Transcriptomic stratification of GBM reveals progressive tumor and recurrence-adapted states.** Differential gene expression analysis identified robust transcriptional signatures distinguishing GBM tumors from normal brain tissue and recurrent tumors from primary GBMs. These signatures were used to construct two composite metrics: a TS capturing oncogenic transformation and an RS reflecting transcriptional programs associated with tumor adaptation and relapse. Initial stratification using the TS separated samples into a normal-like group (Class 1) and tumor-like groups (Classes 2–4). Class 1 samples exhibited low TS and retained neuronal and synaptic gene expression programs, consistent with non-neoplastic brain tissue. In contrast, tumor samples showed uniformly high TS, reflecting activation of cell cycle, proliferation, and metabolic pathways (Table 4).

Further stratification of tumor samples using the RS revealed three distinct tumor states. Class 2 (Primary Tumor- Proliferative Core) displayed high TS but low RS and was characterized by strong upregulation of cell cycle regulators and mitotic genes, consistent with classical proliferative GBM biology. Class 3 (Primary Tumor-Transitional) showed intermediate RS and a mixed transcriptional phenotype, combining residual proliferative signals with emerging extracellular matrix and immune-related gene expression, suggesting early adaptive remodeling. Class 4 (Recurrence-Adapted Aggressive tumors) exhibited high TS and high RS, marked by immune-related chemokines, extracellular matrix components, and loss of proneural lineage markers, indicative of advanced tumor plasticity and recurrence-associated adaptation. Together, this hierarchical classification captures a continuum from normal brain tissue through primary tumor growth to highly adaptive, recurrence-associated GBM states.

The proposed four-class gene expression-based classification provides a practical framework for translating transcriptomic heterogeneity of glioblastoma into clinically interpretable tumor states. By reducing genome-wide expression data to compact gene signatures and composite scores, this approach enables stratification beyond conventional histopathology while remaining compatible with simplified molecular assays.

## 4. Discussion

The bioinformatics analysis reveals that, while GBM, both primary and recurrent, shares pathways related to cancer and organismal injury, tumor recurrence is accompanied by distinct molecular changes, particularly in immune response, metabolism, and systemic effects on other organ systems. These findings suggest that recurrent GBM may exploit different biological mechanisms. A limitation of this study is the unequal sample size across groups, particularly the relatively limited number of recurrent GBM and non-neoplastic brain samples compared with primary tumors. This imbalance largely reflects the availability of high-quality, well-annotated datasets in TCGA, in which recurrent GBM and normal brain tissues are underrepresented due to clinical and ethical constraints associated with tissue collection [18]. To address this limitation, our analyses emphasized pathway- and gene set-level changes rather than individual gene-level differences, as pathway-based approaches are generally more robust to sample size variability and better capture coordinated biological processes underlying tumor behavior.

Thus, IPA-based disease and function analysis (Table 2) revealed Cancer, Organismal Injury and Abnormalities, and Neurological Diseases as the most enriched categories, underscoring the aggressive oncogenic nature of GBM and its profound disruption of normal neural tissue. Such enrichment of organismal injury-related functions is also associated with widespread tissue damage and remodelling in the tumor microenvironment. Significantly enriched pathways at the cellular level involved in growth, proliferation, movement, and maintenance would be expected, given that GBM cells exhibit highly proliferative and invasive activity and can drive tumor infiltration, recurrence, and therapeutic resistance.

In the recurrent versus normal and recurrent versus primary tumor comparisons, additional enrichment was observed in pathways related to cell signaling, cell-to-cell communication, and inflammatory responses, consistent with the network analyses shown in Figure 5, Figure 6 and Figure 7. These pathways likely support tumor survival, adaptation, and immune evasion in the post-treatment microenvironment, where selective pressure from therapy can favor resistant clones [12]. Notably, the recurrent GBM group exhibited strong activation of immune-related networks, including cytokine signaling and leukocyte trafficking, indicating that tumor recurrence is accompanied by reprogramming of the tumor–immune interface, which may facilitate immune suppression and continued tumor growth [19]. Collectively, these findings suggest that recurrent GBM represents a biologically distinct and more aggressive phenotype, shaped by both intrinsic tumor evolution and microenvironmental remodeling [11,12,13].

Understanding these alterations in gene expression and pathways is essential for developing new targeted therapies and overcoming resistance mechanisms [20]. Several independent transcriptomic and network-based studies in GBM underpin the wider significance of the pathways featured in our analysis. Integrative RNA-seq and proteomics analysis, for example, has been utilized to identify dysregulated surfaceome signatures and immune-associated genes that correlate with clinical outcomes in GBM (e.g., CD44, HLA-DRA) [21]. We used metabolism-specific transcriptomic subtyping also to highlight the contributions of metabolic reprogramming and immune infiltration to the characterization of biologically and clinically distinct GBM subgroups [22]. Studies combining bulk and spatial transcriptomic approaches demonstrate that a diverse cell assembly, metabolism-based changes, and tumor–immune interactions drive the progression and resistance of GBM therapy [23]. Together, the independent studies support the biological significance of the pathways described in our work and add value for further understanding of transcriptomic changes in GBM: additionally, they highlight the utility of multi-cohort and network analysis in elucidating mechanisms underlying malignancy and recurrence [24,25]. Histological-based assessments provide a detailed view of the inflammatory landscape in recurrent GBM. By examining immune cell infiltration and tissue architecture, these analyses reveal key patterns of immune activation, extracellular matrix remodeling, and localized inflammatory responses. Such insights help to characterize the tumor microenvironment in recurrent GBM and may inform the development of strategies targeting immune-related pathways to improve therapeutic outcomes [26].

The Venn diagram and network analysis presented in Figure 9 provide insights into the molecular interconnectivity between EMT, drug resistance, and recurrence in GBM. The Venn diagram highlights a small but significant set of genes shared across these biological categories, suggesting that recurrent GBM evolves through coordinated molecular programs involving invasion, matrix remodeling, and therapy resistance. Among the intersecting genes, IGFBP2 and POSTN are known regulators of GBM invasiveness and angiogenesis, with elevated expression linked to poor prognosis and resistance to radiotherapy [27,28,29,30]. In addition, BIRC5 and MKI67, key markers of proliferation and treatment resistance, which are upregulated in recurrent tumors in this dataset, further indicate that recurrent GBM cells sustain proliferative signalling despite cytotoxic stress [31]. MMP9, a matrix metalloproteinase involved in ECM degradation, further supports the hypothesis that ECM remodeling facilitates tumor invasion and recurrence [32]. The identification of COL1A1, COL3A1, and NNMT as recurrent-specific genes emphasizes the importance of extracellular matrix reorganization and metabolic adaptation in tumor relapse. Collagen-related genes (COL1A1, COL3A1) contribute to structural changes in the tumor microenvironment that enhance invasiveness and therapy evasion [33], while NNMT (nicotinamide N-methyltransferase) is implicated in epigenetic reprogramming and metabolic flexibility [34], both hallmarks of recurrent GBM.

Recurrent tumors exhibit increased intercellular communication within a tumor microenvironment enriched with immune and stromal compartments, which can facilitate immune, inflammatory, and extracellular matrix-related pathways that increase the risk of therapeutic resistance. These findings emphasize the need for recurrence-specific therapeutic strategies, including microenvironment-targeted, immunomodulatory, or combination approaches. The EMT- and metabolism-associated regulators, as well as extracellular matrix components, could also contribute to invasiveness and lead to treatment failure. Drawing on retrospective transcriptomic data, this analysis highlights the importance of recurrence-specific molecular profiling to guide personalized therapeutic approaches.

We propose a gene-expression-based approach that integrates tumorigenic and recurrence-related transcriptional programs to stratify GBM into biologically meaningful states. The TS is a powerful tool that effectively divides neoplastic and non-neoplastic tissue and accounts for central oncogenic features, including cell cycle activation and metabolic rewiring, consistent with those observed in existing GBM signatures. Further delineating tumor heterogeneity, the RS shows that cancer cells undergo progressive transcriptional shifts with recurrence, including immune remodelling, extracellular matrix reorganization, and loss of lineage identity. The switch from proliferative primary tumors (Class 2) to recurrence-adapted aggressive states (Class 4) follows known patterns of GBM evolution, in which both therapeutic pressure and microenvironmental stress drive distinct phenotypic plasticity and immune interactions. This tendency to downregulate proneural markers, combined with enrichment of immune and stromal genes in recurrence-adapted tumors, is consistent with earlier studies showing that recurrence-adapted GBMs adopt mesenchymal-like and immunomodulatory programs linked with poor prognosis and enhanced treatment resistance.

This study is based on unpaired normal, primary, and recurrent GBM samples, as patient-matched longitudinal specimens were not available, limiting within-patient analysis of disease progression. Consequently, Class 3 (transitional) and Class 4 (recurrence-adapted) states represent cohort-level transcriptional programs rather than individual tumor trajectories. Despite substantial inter- and intra-patient heterogeneity in GBM, the consistent emergence of recurrence-associated gene sets supports the biological relevance of these classes as distinct tumor states. Importantly, the identification of transitional and recurrence-adapted classes highlights tumor states associated with treatment adaptation and an increased risk of recurrence. Because the classification framework is score-based rather than strictly categorical, it enables longitudinal monitoring of transcriptional shifts, providing a potential means to track tumor evolution during and after therapy. Although external validation is required, this approach represents a technically sound step toward integrating molecular stratification into GBM research and future clinical applications.

## 5. Conclusions

These data provide a comprehensive transcriptomic framework describing molecular changes associated with GBM progression and recurrence. Through integrative transcriptomic profiling combined with IPA-based pathway analysis, we identified coordinated alterations in gene expression linked to hallmark processes of GBM, including enhanced proliferation, invasion, immune signalling, extracellular matrix remodelling, and metabolic reprogramming. Importantly, many of the pathways and functional categories highlighted in this study (such as inflammatory signaling, immune–tumor interactions, and stress-adaptation mechanisms) are consistent with previously reported drivers of GBM aggressiveness and therapeutic resistance, lending biological plausibility to the predicted gene expression changes. Nonetheless, the present findings should be regarded as hypothesis-generating, as direct functional validation of individual genes and pathways remains necessary.

The integrative analysis further revealed a biologically coherent gene-expression-based stratification of GBM into four molecular classes, capturing progressive transitions from normal brain tissue to primary tumor states and ultimately to a recurrence-adapted phenotype. Beyond distinguishing tumor from non-tumor tissue, this classification highlights the intrinsic heterogeneity of GBM, including a transitional tumor state and a recurrence-associated program characterized by immune interaction, extracellular matrix remodelling, and loss of proneural lineage features. These observations support a dynamic model of GBM evolution driven by transcriptional plasticity rather than fixed molecular identities.

The derived minimal gene signatures offer a practical framework for sample stratification and provide a foundation for future studies integrating redox-regulated processes, miRNA-mediated regulation, and adhesion-related mechanisms. However, extensive experimental validation (using independent patient cohorts, functional assays, and in vivo models) is required to confirm the biological roles of these predicted genes and pathways. Future research should also integrate multi-omics approaches to refine these molecular classes and assess their clinical utility. Collectively, these efforts may ultimately inform precision medicine strategies aimed at improving therapeutic response and limiting recurrence in patients with GBM.

## Figures and Tables

**Table 1:** Clinical data for the GBM patients included in the study.

		Normal Tissue—TN	Tumor Tissue—TT	Recurrent Tissue—TR
		5	154	13
Gender	Female	3	54	6
	Male	2	100	7
Age	10–19	0	1	1
	20–29	0	4	3
	30–39	0	13	4
	40–49	2	24	2
	50–59	3	39	2
	60–69	0	48	1
	70–79	0	18	0
	80–89	0	7	0
Histological type	Untreated primary (de novo) GBM	4	132	10
	Treated primary GBM GBM Multiforme (GBM)	1 0	12 10	3 0

**Table 2:** Diseases and disorders, respectively, molecular and cellular functions altered in the analysed GBM subgroups.

Name	Analysed Group	Biological Process	*p*-Value Range	# Number of Molecules
Diseases and Disorders	Tumor versus normal	Cancer	4.65 × 10^−13^–0.00 × 10^−00^	5541
		Organismal Injury and Abnormalities	6.95 × 10^−13^–0.00 × 10^−00^	5587
		Gastrointestinal Disease	1.96 × 10^−13^–4.69 × 10^−292^	5121
		Endocrine System Disorders	4.65 × 10^−13^–9.06 × 10^−284^	4923
		Neurological Disease	6.95 × 10^−13^–1.21 × 10^−204^	4340
	Recurrent versus normal	Cancer	3.55 × 10^−13^–0.00 × 10^−00^	4346
		Endocrine System Disorders	3.55 × 10^−13^–0.00 × 10^−00^	3910
		Gastrointestinal Disease	2.35 × 10^−13^–0.00 × 10^−00^	4041
		Organismal Injury and Abnormalities	3.58 × 10^−13^–0.00 × 10^−00^	4385
		Neurological Disease	3.28 × 10^−13^–1.15 × 10^−239^	3461
	Recurrent versus tumor	Cancer	1.41 × 10^−2^–5.13 × 10^−13^	132
		Organismal Injury and Abnormalities	1.41 × 10^−2^–5.13 × 10^−13^	133
		Dermatological Diseases and Conditions	1.33 × 10^−2^–1.76 × 10^−11^	111
		Gastrointestinal Disease	1.41 × 10^−2^–1.46 × 10^−10^	124
		Inflammatory Response	1.34 × 10^−2^–1.46 × 10^−10^	58
Molecular and Cellular Functions	Tumor versus normal	Cellular Assembly and Organization	5.50 × 10^−13^–2.01 × 10^−83^	1566
		Cellular Function and Maintenance	6.34 × 10^−13^–2.01 × 10^−83^	2130
		Cellular Movement	6.81 × 10^−13^–3.09 × 10^−66^	1780
		Cellular Development	1.33 × 10^−13^–8.64 × 10^−66^	2322
		Cellular Growth and Proliferation	1.33 × 10^−13^–8.64 × 10^−66^	2240
	Recurrent versus normal	Cellular Assembly and Organization	3.21 × 10^−13^–2.80 × 10^−74^	1347
		Cellular Function and Maintenance	2.29 × 10^−13^–2.80 × 10^−74^	1904
		Cellular Development	2.29 × 10^−13^–2.30 × 10^−71^	1852
		Cellular Growth and Proliferation	2.29 × 10^−13^–2.30 × 10^−71^	1787
		Cell Morphology	1.02 × 10^−13^–2.10 × 10^−59^	1202
	Recurrent versus tumor	Cellular Movement	1.26 × 10^−2^–1.77 × 10^−8^	48
		Cell-To-Cell Signalling and Interaction	1.41 × 10^−2^–1.10 × 10^−6^	38
		Cell Signalling	1.21 × 10^−2^–2.42 × 10^−5^	23
		Molecular Transport	1.21 × 10^−2^–2.42 × 10^−5^	43
		Vitamin and Mineral Metabolism	1.21 × 10^−2^–2.42 × 10^−5^	26

**Table 3:** The top four associated networks for the analysed GBM groups.

Analyses Group	Top Diseases and Functions	Score	Focus Molecules
Tumor versus normal	N1: Developmental Disorder, DNA Replication, Recombination, and Repair, Gene Expression	30	34
	N2: Cancer, Organismal Injury and Abnormalities, Respiratory Disease	30	34
	N3: Embryonic Development, Nervous System Development and Function, Organismal Development	30	34
	N4: Cancer, Hematological Disease, Immunological Disease	30	34
Recurrent versus normal	N1: Cell-mediated Immune Response, Cellular Development, Cellular Function and Maintenance	31	33
	N2: Amino Acid Metabolism, Molecular Transport, Small Molecule Biochemistry	31	33
	N3: Cardiovascular Disease, Congenital Heart Anomaly, Developmental Disorder	31	33
	N4: Cancer, Gastrointestinal Disease, Organismal Injury and Abnormalities	31	33
Recurrent versus tumor	N1: Gastrointestinal Disease, Ophthalmic Disease, Organismal Injury and Abnormalities	32	16
	N2: Neurological Disease, Organismal Injury and Abnormalities, Psychological Disorders	32	16
	N3: Dermatological Diseases and Conditions, Immunological Disease, Inflammatory Disease	29	15
	N4: Cellular Movement, Hematological System Development and Function, Immune Cell Trafficking	29	15

**Table 4:** Gene expression-based classification with biological interpretation and marker genes.

Class	Name	TS	RS	Biological Interpretation	Representative Upregulated Genes	Representative Downregulated Genes
**Class 1**	**Normal-like**	Low	Not applicable	Preserved neuronal/synaptic programs; absence of oncogenic activation	GRIN1, PRKCG, RYR2, GABRA5, SLC17A7, NEFM, SYN2, C1QL3	MYBL2, UBE2C, TOP2A, CCNB2, BIRC5
**Class 2**	**Primary Tumor—Proliferative Core**	High	Low	Canonical tumor biology dominated by cell cycle, proliferation, and metabolic rewiring	MYBL2, UBE2C, TOP2A, RRM2, PBK, DLGAP5, CCNB2, BIRC5	GRIN1, PRKCG, GABRA5, NEFM
**Class 3**	**Primary Tumor—Transitional**	High	Intermediate	Mixed phenotype with partial loss of lineage identity and emerging immune/ECM signaling	MYBL2, TOP2A, RRM2 (moderate); HAS1, COL6A3 (emerging)	OLIG1, OLIG2, BCAN (partial loss)
**Class 4**	**Recurrence-Adapted (Aggressive)**	High	High	Immune–ECM remodeling, stress adaptation, and reduced proneural lineage markers	CCL18, CCL13, CD209, CR1, LILRB5, HAS1, COL6A3, LYVE1, PAPPA	OLIG1, OLIG2, BCAN, ARC, HES6, SLITRK3

## Data Availability

The data presented in this study are available on request from the corresponding author.

## Supplementary Materials

The following supporting information can be downloaded at: https://www.mdpi.com/article/10.3390/medicina62020336/s1, Table S1 Altered gene expression in primary tumor versus normal brain tissue, Table S2 Altered gene expression in recurrent tumor versus normal brain tissue, Table S3. Altered gene expression in recurrent tumor versus primary tumor.

## Abbreviations

The following abbreviations are used in this manuscript:

ECM	Extracellular Matrix
EMT	Epithelial–mesenchymal transition
FC	Fold change
FDR	False discovery rate
GBM	Glioblastoma multiforme
IPA	Ingenuity Pathway Analysis
*p*-value	Probability value
PCA	Principal component analysis
PPI	Protein–protein interaction
RS	Recurrence score
RNA	Ribonucleic acid
TCGA	The Cancer Genome Atlas program
TN	Normal tissue
TR	Recurrent tissue
TS	Tumor score
TT	Tumor tissue
